# Topical Delivery of Carvedilol Loaded Nano-Transfersomes for Skin Cancer Chemoprevention

**DOI:** 10.3390/pharmaceutics12121151

**Published:** 2020-11-27

**Authors:** Mengbing Chen, Md Abdullah Shamim, Ayaz Shahid, Steven Yeung, Bradley T. Andresen, Jeffrey Wang, Vijaykumar Nekkanti, Frank L. Meyskens, Kristen M. Kelly, Ying Huang

**Affiliations:** 1 Department of Pharmaceutical Sciences, College of Pharmacy, Western University of Health Sciences, Pomona, CA 91766, USA; mengbing.chen@westernu.edu (M.C.); mdabdullah.shamim@westernu.edu (M.A.S.); ashahid@westernu.edu (A.S.); skyeung@westernu.edu (S.Y.); bandresen@westernu.edu (B.T.A.); jwang@westernu.edu (J.W.); nekkanti_vk@yahoo.com (V.N.); 2 Departments of Medicine and Biological Chemistry, Chao Family Comprehensive Cancer Center, University of California, Irvine, CA 92868, USA; flmeyske@hs.uci.edu; 3 Department of Dermatology, University of California, Irvine, CA 92697, USA; kmkelly@hs.uci.edu

**Keywords:** β-blocker, carvedilol, ultraviolet, skin cancer, chemoprevention, transfersome, topical delivery

## Abstract

The β-blocker carvedilol has been shown to prevent skin carcinogenesis in vitro and in vivo. Since systemic absorption of the β-blocker may cause cardiovascular disturbance, we developed a carvedilol loaded transfersome for skin-targeted delivery. Transfersomes were prepared using phospholipids and surfactants at various ratios and characterized. One formulation (F18) selected for further analysis was composed of carvedilol, soy phosphatidylcholine, and Tween-80 at a ratio of 1:3:0.5, which had a particle size of 115.6 ± 8.7 nm, a zeta potential of 11.34 ± 0.67 mV, and an encapsulation efficiency of 93.7 ± 5.1%. F18 inhibited EGF-induced neoplastic transformation of mouse epidermal JB6 P+ cells at non-toxic concentrations, while only high concentrations induced cytotoxicity in JB6 P+ and human keratinocytes HaCaT. Compared to the free drug, F18 released through the dialysis membrane and permeated through the porcine ear skin at a slower rate, but similarly depositing the drug in the epidermis and dermis of the skin. Consistently, surface application of F18 on reconstructed full-thickness human skin showed slower drug permeation, while it suppressed ultraviolet-induced DNA damage, inflammatory gene expression, and apoptosis. These data indicate that transfersome is a promising topical delivery system of carvedilol for preventing ultraviolet-induced skin damage and carcinogenesis.

## 1. Introduction

Skin cancer, consisting of basal- and squamous-cell carcinoma (non-melanoma skin cancer) and melanoma, is the most common type of cancer in the U.S. and the world [1]. Solar ultraviolet (UV) radiation, mainly consisting of UVA (320,400 nm) and UVB (290,320 nm), increases the risk of skin cancer [2]. There is a strong need to develop agents to inhibit and reverse UV-induced biochemical changes that could lead to skin carcinogenesis. Previous studies have indicated that the β-blocker carvedilol, commonly used to manage cardiovascular disorders such as hypertension and heart failure, showed promising activity in preventing chemical carcinogen and UV-induced skin carcinogenesis in vitro and in vivo [3,4]. The anticancer mechanisms for carvedilol involve multifunctional action including attenuating UV-induced oxidative stress, DNA damage, inflammation, and oncogenic signaling pathways [4,5]. Further supporting a role of carvedilol in cancer prevention, a population-based cohort study of 6771 individuals demonstrated that long-term use of carvedilol was associated with a reduced risk of several types of cancer [6].

Although both topical and oral administration of carvedilol showed inhibitory activity against chemical carcinogen-induced H-RAS mutation and skin hyperplasia in mice, topical application of carvedilol dissolved in acetone was more effective [3]. In addition, topical application of carvedilol dissolved in acetone prevented acute UV-induced skin damage and chronic UV-induced skin tumor formation in hairless mice [3,4]. Importantly, as carvedilol is a highly potent β-blocker with an IC_50_ ~1 nM, repurposing to a cancer preventive agent faces obstacles because oral administration may cause undesirable cardiovascular side effects such as bradycardia and hypotension. Topical administration is an important alternative route for skin cancer prevention because the drug will be more likely to reach the sites of damage and is an easy and widely used method of self-treatment. Carvedilol is a highly lipophilic compound with low molecular weight and a favorable logarithmic partition coefficient (log *p* value 3.8), making it suitable for skin-specific delivery. Since carvedilol is insoluble in water, acetone is used as a vehicle and skin penetration enhancer for topical administration in animal models. Although acetone is a commonly used vehicle for skin cancer animal studies, the use of acetone is not recommended for humans because it dehydrates and dries the skin excessively. In addition, since the use of acetone disrupts the barrier function of the skin [7], it may cause unwanted systemic absorption of topical drugs. Therefore, a suitable topical delivery system is required in order to repurpose carvedilol into a topical skin cancer chemopreventive agent.

A variety of nanoparticle-based technologies have been examined for their topical delivery efficacy in various skin disorders such as skin cancer [2,8,9] including polymers, liposomes, amphiphilic cyclodextrins, dendrimers, gold nanoparticles, micelles, carbon nanotubes, and quantum dots. Among these nanoparticles, the ultra-deformable vesicular system (also named flexible liposomes or “transfersomes” by the inventors; transfersome is the term used throughout this report), which are edge activator-based nanocarriers, showed enhanced drug penetration through the intercellular lipid matrix by blending with the stratum corneum (SC) and modifying the lipid lamellae [2,8,9,10,11]. Furthermore, transfersome can effectively protect the drug against undesired absorption into cutaneous blood vessels and are capable of retaining the drug in the skin [9,11]. In contrast, conventional liposomes have been shown with less value as topical carriers because they do not deeply penetrate the skin, but rather remain confined to upper layers of the SC [8].

The present study was aimed at developing a novel topical delivery system of carvedilol for skin cancer prevention using transfersome as the nanocarrier. Transfersomes with various ratios of carvedilol, phospholipid, and edge activator were prepared and characterized. One transfersomal formulation, F18, which showed relatively small particle size, high encapsulation efficiency, and stability, was selected, optimized, and further characterized. Although F18 showed slower permeation through the porcine ear skin and reconstructed full-thickness human skin, it demonstrated a similar degree of protection against UV-induced skin damages as the free drug dissolved in acetone. These results, for the first time, demonstrate the feasibility of using transfersome as a topical delivery system for carvedilol.

## 2. Materials and Methods

### 2.1. Compounds

Carvedilol was purchased from TCI America (Portland, OR, USA). Tween-80 and sodium cholate were purchased from VWR (Radnor, PA, USA). l-α-phosphatidylcholine (Soy PC or SPC), l-α-phosphatidylcholine, hydrogenated (Hydro Egg PC or HEPC), and 1,2-distearoyl-sn-glycero-3-phosphocholine (DSPC) were purchased from Avanti Polar Lipids, Inc. (Alabaster, AL, USA).

### 2.2. Preparation of Carvedilol Loaded Transfersomes

Carvedilol loaded transfersomes were prepared using a thin-film hydration method, as described in previous reports [12,13]. Briefly, lipids, surfactants, and carvedilol were solubilized in chloroform:methanol (2:1, *v*/*v*) in a round bottom flask and mixed well. The organic solvent was gradually removed under reduced pressure in a rotary evaporator at 45 °C for 30 min until a thin film was formed. The thin film was kept in a desiccator overnight to completely remove the residual solvent before it was hydrated with phosphate-buffered saline (PBS) (pH 7.4) at the transition temperature (51 °C) of the lipid components using a rotary evaporator for 30 min. For selected formulation F18 and the control transfersome without drug loading (plain transfersome), after the suspension was sonicated in a bath for 5 min, it was extruded through a 100 nm pore size membrane (Avanti Polar Lipids, Alabaster, AL, USA) through an extruder (Liposofast LF-50, Avestin, Ottawa, ON, Canada) for five cycles to obtain the final formulations.

### 2.3. Determination of Particle Size and Zeta Potential

The transfersomes were dispersed in PBS or nano-pure water for particle size and zeta potential determination, respectively. Particle sizes were analyzed by using a Nanobrook Omni particle sizer (Brookhaven Instruments Corporation, Holtsville, NY, USA), and zeta potential was determined using a Nano ZS90 Zetasizer (Malvern Panalytical, Malvern, United Kingdom). For stability evaluation, the suspensions were stored at 4 °C, and the size was measured every two to three days for up to 49 days (*n* = 3 for plain transfersomes; *n* = 6 for F18).

### 2.4. Determination of Drug Encapsulation Efficiency (EE)

Carvedilol loaded transfersomes were centrifuged in 30,000-dalton cutoff Nanosep^®^ tubes at 14,000 rpm for 1 h at 4 °C. Free carvedilol concentration in the filtrate was analyzed via HPLC. To measure the total concentration of carvedilol, the transfersome suspension (10 uL) was mixed with 990 uL of methanol and vortexed for 1 h to disrupt the transfersomes. The encapsulation efficiency was calculated by the formula below.

$$\%\;\mathrm{encapsulation}\;\mathrm{efficiency}=\;\frac{\left(\mathrm{total}\;\mathrm{carvedilol}\;\mathrm{concentration}-\mathrm{free}\;\mathrm{carvedilol}\;\mathrm{concentration}\right)}{\mathrm{total}\;\mathrm{carvedilol}\;\mathrm{concentration}}\;\times \;100$$

### 2.5. HPLC Analysis

An Agilent 1260 HPLC system (Agilent Technologies Inc., Santa Clara, CA, USA) was used for the detection of carvedilol drug levels, equipped with a quaternary pump (G1311B), an autosampler (G7129A), an automatic thermostatic column compartment, a DAD detector (G1315D), and a computer with Agilent OpenLAB CDS Chemstation Edition for LC&LC/MS Systems (Rev C.01.07) for the analysis of the HPLC data. Carvedilol was separated on a BDS Hypersil C18 reverse-phase column (2.1 × 150 mm; 2.4 um) (Thermo Scientific, Waltham, MA, USA) together with a C18 guard column (10 mm × 2.1, 3 um) (Thermo Scientific) with the column temperature set at 35 °C. The mobile phase was a mixture of acetonitrile:buffer (38:62), the buffer was made of 20 mM ammonium acetate, 0.1% triethylamine, and adjusted to pH 4.5 with phosphoric acid. The flow rate was 0.2 mL/min. Carvedilol was analyzed at the wavelength of 240 nm. One μg/mL of propranolol was used as an internal standard (retention time for carvedilol: 6.4; propranolol: 3.9)

### 2.6. In Vitro Drug Release Study

The in vitro drug release study was carried out using the Pur-A-Lyzer Mini Dialysis Kit and the molecular weight cut off (MWCO) of the dialysis membrane was 3.5 Kda (Sigma-Aldrich, St. Louis, MO, USA). The study was performed in a shaking incubator at 100 rpm and 37 °C. The carvedilol loaded transfersome suspension containing 0.2 mg carvedilol or the same amount of carvedilol in acetone (the acetone volume was the same as the suspension) was added into the dialysis tubes. The dialysis tubes were immersed in 200 mL of PBS (pH 7.4) as the release medium. At predetermined time intervals (0.5, 1, 2, 3, 4, 6, 8, 24 h), 1 mL samples were withdrawn from the release medium and replaced with 1 mL of fresh PBS. The samples were then analyzed via the HPLC method.

### 2.7. Ex Vivo Skin Permeation Study

The ex vivo skin permeation studies were performed using Franz diffusion cells (Crown Glass Company, Somerville, NJ, USA) and porcine ear skin. The ears were purchased from Sierra for Medical Science (Whittier, CA, USA). The outer surface of the skin was excised using a surgical scalpel from the cartilage, with the adipose subcutaneous tissue removed, then placed in zip bags, stored at −80 °C, and used within one year. The day before conducting the permeation study, the skin was thawed overnight at 4 °C. Integrity of the skin was examined using Tewameter^®^ TM300 (C+K GmbH, Cologne, Germany) right before use in experiments. Only skin with transepidermal water loss (TEWL) < 15 g/h/m^2^ was used. The skin was sandwiched between donor and receptor compartments of the diffusion cell with the stratum corneum layer facing the donor compartment. The receptor compartment was filled with 5.5 mL of pH 7.4 PBS and maintained at 37 °C under magnetic stirring. The carvedilol loaded transfersome suspension 0.2 mL containing 4 µg carvedilol or the same amount of carvedilol in acetone was applied to the porcine skin in the donor compartment, and the donor compartments were then covered with parafilm to avoid extra evaporation. At predetermined time intervals (0.5, 1, 2, 3, 4, 6, 20, 24 h), 0.2 mL PBS in the receptor compartment was collected and replaced with 0.2 mL fresh PBS. The drug content in the samples were analyzed by HPLC and the concentration was corrected for sampling effects according to the reported method [14]. To analyze the drug penetration into the skin layers, a tape-stripping method was used according to reports [15,16]. Briefly, the first tape with the residue of drugs from the skin surface was discarded. Ten additional pieces of adhesive tape (3M, Scotch, St Paul, MN, USA) were then applied to the skin to remove the stratum corneum layer. After removing the stratum corneum, the remaining layers consisting of the epidermis and dermis were collected in 1 mL of 50% methanol. The samples were then mechanically shaken for 1 h and homogenized for at least 1 min to extract the drug.

### 2.8. Cell Lines and Cytotoxicity Assay

JB6 CI 41-5a (JB6 P+), which is a mouse epidermal cell line sensitive to the promotion of transformation, was purchased from American Type Culture Collection (ATCC, Manassas, VA, USA). JB6 P+ cells were maintained in Eagle’s minimum essential medium (EMEM) containing 4% heat-inactivated fetal bovine serum and 1% penicillin/streptomycin. HaCaT cells, an immortalized, non-tumorigenic human keratinocyte cell line, were a gift from Dr. Peter Fu at the National Center for Toxicological Research, U.S. Food and Drug Administration. HaCaT cells were maintained in Dulbecco’s modified Eagle’s medium (Invitrogen Life Technologies, Carlsbad, CA, USA) supplemented with 10% fetal bovine serum and 1% penicillin/streptomycin. Both cell lines were cultured at standard cell culture conditions (37 °C, 5% CO_2_ in a humidified incubator). Ninety-six-well plates were seeded with 3000 to 4000 cells per well and allowed to attach overnight. Cells were treated with test compounds for 72 h at 37 °C in 5% CO_2_/95% air. Cell viability was determined using the sulforhodamine B (SRB) assay (Sigma-Aldrich, St. Louis, MO, USA) according to the manufacturer’s protocols.

### 2.9. Intracellular Uptake of Transfersomes

The transfersomes (F18) were prepared as described above, but with the addition of 1 mol % DiI (ThermoFisher) to the initial mixture of lipids. For cellular uptake assay, JB6 P+ cells were seeded in a 6-well plate and allowed to attach to the plate overnight at 37 °C. F18 containing 10 μM (4 µg/mL) carvedilol, with or without being labeled with DiI, was incubated with the cells for 1, 3, 5, and 24 h. The cells were rinsed with PBS, and images were taken using an EVOS Cell Imaging System fluorescence microscope (ThermoFisher). The same experiment was repeated by seeding the cells on a 35 mm cell imaging dish with a glass bottom (ibidi GmbH, Gräfelfing, Germany), and cells were incubated with F18 for 3 h. After the cells were rinsed with PBS, the nuclei were stained with 5 µM Hoechst 33342 nucleic acid stain (ThermoFisher) for 20 min at 37 °C. The cells were then rinsed with PBS and observed using a Leica TCS SPE confocal microscope (Leica, Buffalo Grove, IL, USA) with an HC PL APO CS2 40× 1.10 NA water immersion objective. Hoechst 33342 was observed with a 405 laser at 10% power collecting emitted light between 410 nm and 476 nm with 901.6 v gain and −1.1% offset. The Hoechst signal was pseudocolored blue. DiI was observed with a 532 laser at 25% power collecting emitted light between 542 nm and 700 nm with 970.6 v gain and −8.4% offset. The DiI signal was pseudocolored green. Images were collected as Z-stacks to capture the entire cell; an optimal slice number for the stack size was determined by the Leica software using the Nyquist theorem. The Z-stacks were displayed using the Lecia software; the background was set to black and intensity to 200 (default settings), and the blue and green channels were set to an opacity of 50; minimum of 9 and 25, respectively; maximum of 75 and 100, respectively; and gamma of 0.75. The images are displayed obliquely and isometrically.

### 2.10. UV Light Source

UV lamps emitting UVB (280–320 nm; 54% of total energy), UVA (320,400 nm; 37% of total energy), UVC (100,280 nm; 2.0% of total energy), and visible light (400,450 nm; 7.0% of total energy) (UVP, Upland, CA, USA) were used. Stable power output (mW/cm^2^) was measured using a UVX radiometer (UVP) coupled with a sensor with a calibration point of 310 nm (UVX-31, UVP), and exposure time was calculated using the following formula: dose (mJ/cm^2^) = exposure time (s) × output intensity (mW/cm^2^). Quality control of the lamps and exposure time were calculated and monitored before each use of the lamps to account for changes in power output over time.

### 2.11. 3Dimensional (3D) Human Reconstituted Skin Model

EpiDerm™ FT-400 (EFT-400) in a six-well plate was purchased from MatTek Corporation (Ashland, MA, USA). As per the protocols provided by the manufacturer, the tissue was equilibrated in media provided overnight at 37 °C and 5% CO_2_ prior to drug treatment and UV radiation. The dermal side of the tissue was in contact with the EFT-400 media, and the stratum corneum side was exposed to the air. Twenty-four hours before UV treatment, a total dose of 0.8 or 4 μg of free carvedilol in acetone or F-18 (0.1 mL) was applied to the stratum corneum side of the tissue. At predetermined time intervals (0.5, 1, 2, 3, 4, 6, 20, and 24 h), 100 μL of media from each well was collected for HPLC analysis and replaced with 100 uL fresh media. Twenty-four hours later, the drugs on the skin surface were washed with PBS to remove any drugs that were not absorbed, and the tissues were then exposed to 300 mJ/cm^2^ of UV. Immediately following radiation, the same drug doses were applied to the stratum corneum side of the tissue. The tissues were further incubated for 24 h before harvest for RNA, DNA, and histological analysis.

### 2.12. Slot Blot Analysis for Cyclobutane Pyrimidine dimer (CPD) and Pyrimidine (6-4) Pyrimidone Photoproducts (6-4PP)

Genomic DNA was isolated from the human reconstituted skin by using the QIAamp DNA Mini Kit (Qiagen, Redwood City, CA, USA). The DNA samples (500 ng), denatured by boiling in a buffer of 0.4 M NaOH and 10 mM EDTA (for CPD) or water (for 6-4PP) for 10 min, were vacuum-transferred to a nitrocellulose membrane (0.45 Micron, Thermo Scientific) using a Bio-Dot SF microfiltration apparatus (Bio-Rad, Hercules, CA, USA). The DNA damage markers were detected by immunoblot using anti-CPD monoclonal antibodies (Kamiya, Seattle, WA, USA) or using the anti-6-4PP (Cosmo Bio USA, Carlsbad, CA, USA). After the immunoblot, total DNA amounts were visualized by Anti-DNA antibodies, single-stranded, clone 1619 (Sigma-Aldrich), and these values were used to normalize the CPD and 6-4PP values.

### 2.13. Quantitative RT-PCR Analysis

Total RNA was isolated from the skin culture using an RNeasy Mini Kit (Qiagen, Hilden, Germany). cDNA was obtained with the high capacity cDNA Reverse Transcriptase Kit (Thermo Fisher). cDNA and PerfeCTa SYBR Green Supermix (Quanta Biosciences, Inc., Beverly, MA, USA) were combined with primers for human IL-1β, TNF-α, COX-2, and GAPDH (primer sequences: TNF-α 5’-TCAGCTTGAGGGTTTGCTAC-3’ and 5’-TGCACTTTGGAGTGATCGG-3’; COX-2 5′-CCTGGCGCTCAGCCATAC-3′ and 5′-GGTACAATCGCACTTATACTGGTCAA-3′; IL-1β 5′-GGGCCTCAAGGAAAAGAATC-3′ and 5′-AGCTGACTGTCCTGGCTGAT-3′; GAPDH 5′-GGTGAAGGTCGGAGTCAACGG-3′ and 5′-GGTCATGAGTCCTTCCACGAT-3′). Real-time quantitative PCR was performed on a CFX96 Real-Time Thermal Cycler Detection System (Bio-rad, Hercules, CA, USA) and analyzed with the 2^−ΔΔct^ with GAPDH as the normalization control.

### 2.14. Histological Analysis and Terminal Deoxynucleotidyl Transferase dUTP Nick End Labeling (TUNEL) Assay

According to the protocol provided by the manufacturer, the human reconstituted skin was fixed in 10% neutral buffered formalin overnight, dehydrated, paraffin-embedded, sectioned using a microtome, and stained with hematoxylin and eosin (H&E). The unstained deparaffinized slides were used for the transferase dUTP nick end labeling (TUNEL) assay with the DeadEnd™ Colorimetric TUNEL System based on the manufacturer’s protocol (Promega, Madison, WI, USA). Briefly, paraffin-embedded tissue sections were washed in xylene for 5 min, followed by immersion in 100% ethanol for 5 min, rehydrated in decreasing concentrations of ethanol (100–50%) for 3 min, and washed by immersion in 0.85% sodium chloride and phosphate buffer saline (PBS) for 5 min each. Apoptosis was detected by fixing slides in 4% paraformaldehyde in PBS for 15 min, addition of proteinase K solution, and incubation at room temperature for 10–30 min. Equilibration buffer was added to equilibrate at room temperature for 5–10 min, followed by the addition of recombinant terminal deoxynucleotidyl transferase (rTdT) reaction mixture to the tissue sections on the slides and then incubated for 60 min at 37 °C. The reaction was stopped by immersion in 2× saline-sodium citrate (2× SSC) buffer for 15 min, then blocked by the immersion of slides in 0.3% hydrogen peroxide for 3–5 min. Streptavidin horseradish peroxidase (HRP) was added to slides, incubated for 30 min at room temperature, stained with diaminobenzidine (DAB), and developed until a light brown background appeared. The slides were evaluated with a microscope at 10× magnification (Leica DM750, Buffalo Grove, IL, USA).

### 2.15. Statistical Analysis

Data were expressed as a mean ± standard deviation (SD) or standard error (SE) unless stated otherwise. All plots were made using GraphPad Prism version 7.0 (La Jolla, CA, USA), and statistical analysis was conducted using NCSS 2007 (Kaysville, UT, USA). The specific tests are detailed in the text and figure legends. For all statistical analyses, means were indicated to be statistically different when *p* < 0.05.

## 3. Results

### 3.1. Formulation Preparation, Characterization, and Optimization

A pilot study was conducted with a lower final concentration of carvedilol in the formulation suspension (0.1 mg/mL) to identify optimal components and ratio for the transfersomes. Various ratios of carvedilol, phospholipids, and surfactants (Tween-80 or sodium cholate) were combined. The phospholipids (DSPC, SPC, and HEPC) have varying acyl chain length and degree of saturation. SPC is a commonly used natural lipid with an unsaturated acyl chain, and its structure is similar to that of biomembrane phospholipids [17]. HEPC and DSPC both contain saturated acyl chains that can prevent oxidation, while DSPC has longer acyl chains. The surfactant (or edge activator) is the critical component that contributes to the high deformability of the transfersomes by increasing lipid bilayer flexibility and permeability [10]. Tween-80 is a nonionic surfactant that can increase the stability of liposomes [18]. Sodium cholate is an ionic surfactant that can also stabilize liposomes [19].

Thirty formulations were characterized in terms of particle size, polydispersity index (PDI), zeta potential, and encapsulation efficiency (EE) (Table 1). All formulations showed EE% >90%. The zeta potential appears to correlate with the phospholipid type in the transfersome: DSPC and HEPC produced negative zeta potential while SPC produced positive zeta potential. Although many factors may contribute to the efficacy of drug delivery, particle size is one of the most important. According to previous studies, for delivering drugs into the deeper layers of skin, the suitable size should be 300 nm or below [20]. Thus, we used particle size as the first criterion for screening. As can be seen in Table 1, when formulations were made from DSPC and Tween-80, the smallest average size was 246.3 ± 10.8 nm at the ratio of 1:3:0.5 for carvedilol:lipid:surfactant (w:w:w) (F8). When formulations were made from SPC and Tween-80, the smallest average size was 162.9 ± 9.6 nm at the ratio (carvedilol:lipids:surfactant, w:w:w) of 1:3:0.5 (F18). When formulations were made from HEPC and Tween 80, the smallest size was 214.4 ± 5.3 nm at the ratio (carvedilol:lipids:surfactant, w:w:w) of 1:3:0.25 (F21). When formulations were made from SPC and sodium cholate, the smallest size was 189.1 ± 2.2 nm at the ratio (carvedilol:lipid:surfactant, w:w:w) of 1:3:0.5 (F30). Thus, these four candidate formulations (F8, F18, F21, and F30) with the smallest particle size in each of the four groups in Table 1 were selected for further analysis.

Larger scale batches were made for these four selected formulations with higher concentrations of carvedilol (final drug concentration 0.5 mg/mL) and further characterized (Table 2). However, as the concentrations of all components increased, the particle sizes changed. For example, although F21 showed the highest encapsulation efficiency (93.1%), the particle size was too large to penetrate the skin (2164.6 ± 162.5 nm), and therefore, not chosen for further investigation. Among the four formulations, F18 showed the smallest size with 69.7% encapsulation efficiency, and thus was chosen for further studies. New batches of F18 were prepared using carvedilol, SPC, and Tween 80 at the ratio of 1:3:0.5 and applied to an extruder five times in order to further reduce the sizes. The average particle size of resulted F18 was 115.6 ± 8.7 nm, PDI 0.18 ± 0.01, zeta potential 11.34 ± 0.67, and the encapsulation efficiency was 93.7 ± 5.12%. Interestingly, passing through the extruder not only reduced particle size, but also enhanced EE% greatly. F18, the lead transfersomal formulation, was used for further studies.

**Figure 1:**
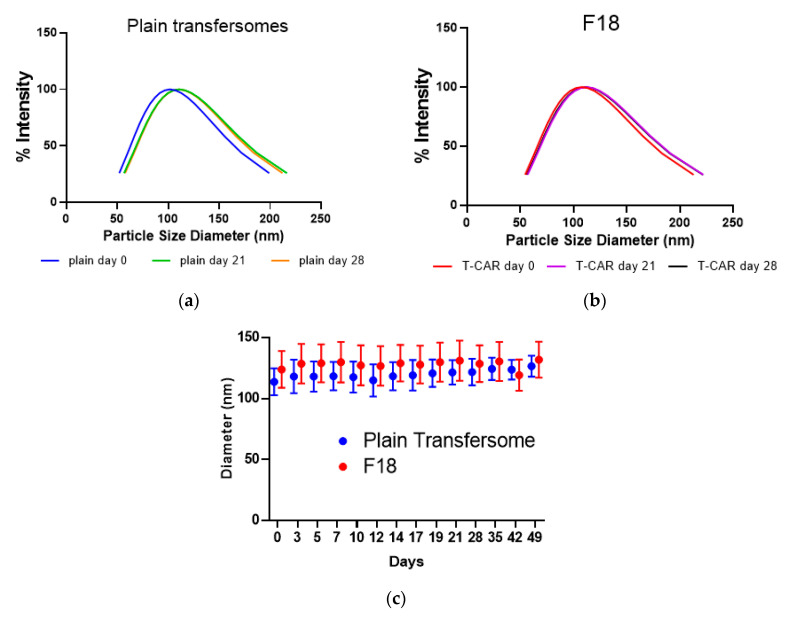
Transfersome size distribution and stability analysis. Representative transfersome size distribution for the plain transfersome (**a**) and the selected transfersome containing carvedilol (F18) (**b**) after storage of different days at 4 °C. (**c**) The size in diameter of the plain transfersomes or F18 as a function of time while maintained at 4 °C (F18 *n*= 6; plain transfersomes *n* = 3). Data expressed as mean ± SD.

Independent batches of F18 and the plain (empty) transfersomes were prepared to determine whether the formulations showed size stability. After passing through the extruder, the plain transfersomes showed an average size of 113.0 ± 10.7 nm, while F18 showed an average diameter of 122.1 ± 13.8 nm (Figure 1a,b). All transfersomes maintained their diameter over the assayed period of 49 days at 4 °C storage condition (Figure 1c), indicating that the formulations are highly stable in terms of particle size.

### 3.2. In Vitro Drug Release Kinetics

**Figure 2:**
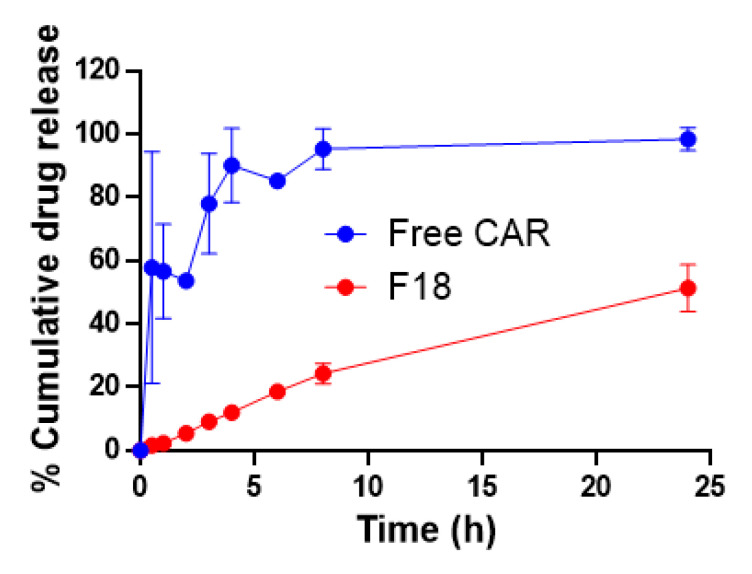
In vitro drug release profiles of free carvedilol dissolved in acetone and carvedilol loaded transfersome F18 (*n* = 3).

The kinetics of drug release from a nanocarrier formulation in vitro is a critical part of the rational design of a drug delivery system as it is a major determinant of the release of the free drug in vivo and the efficacy [21]. For topical drug delivery, the ideal formulation should allow a slower drug release to avoid frequent application and to promote better patient compliance [22]. The drug release profiles in vitro for a total amount of free carvedilol of 0.2 mg in acetone solution and F18 were compared in the release medium of pH 7.4 PBS, mimicking the physiological pH (Figure 2). In the first half-hour, 57% of the carvedilol dissolved in acetone was within the aqueous bath, and within 8 h, carvedilol equilibrated with the aqueous bath. However, F18 showed a much slower drug release profile. At 24 h, the drug release had only achieved 51% of the total drug amount. Repeated-measures ANOVA with the sample as a factor and time as a repeated measure indicated that the two samples were statistically different, *p* = 0.001326 (power = 0.999878). The slower drug release profile may offer an advantage for transfersome as a topical delivery system given that when the formulation is applied to the intact skin, the drug may be delivered at a controlled rate locally, which may reduce the potential for systemic absorption.

### 3.3. Ex Vivo Skin Permeation Study

**Figure 3:**
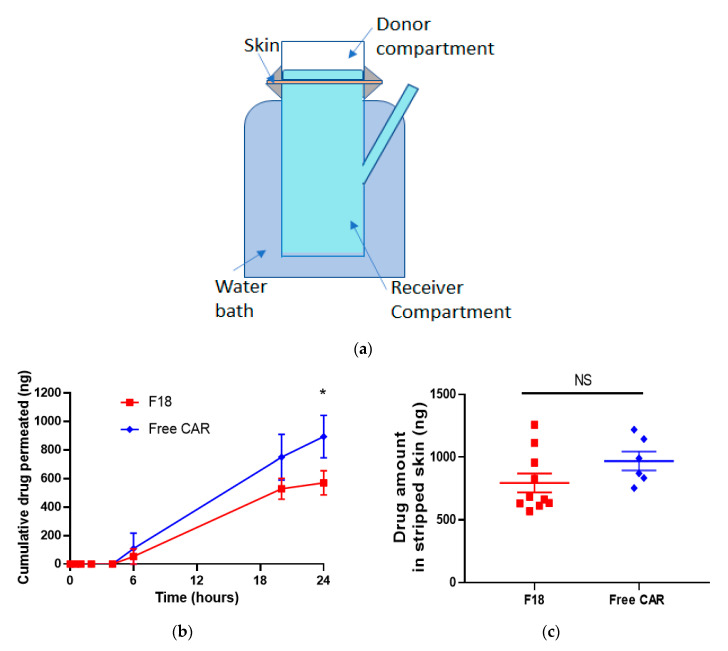
Ex vivo skin drug permeation profiles of free carvedilol and F18. (**a**) Carvedilol acetone solution or F18 (each containing 4.0 μg carvedilol, 0.2 mL) was added to the donor compartment of a Franz diffusion device. 0.2 mL release media (PBS) was withdrawn at predesigned time points for HPLC analysis. (**b**) Cumulative carvedilol permeated into the receptor compartment as a function of time. (**c**) Carvedilol levels in stripped skin (epidermal and dermal layers). After 24 h, the porcine skin was stripped. Drug levels in stripped skin (epidermis and dermis) was determined via HPLC. Data are presented as mean ± SE. (*n* = 10 for F18; *n* = 6 for free drug). * *p* < 0.05 student t-test.

The Franz diffusion cell system was used to determine the skin permeation kinetics for F18 in comparison with free drug dissolved in acetone using porcine ear skin, which is a surrogate for human skin for permeability measurements due to the similarities in skin structure and permeation rate of topically-applied drugs [23] (Figure 3a). The percentage of cumulative carvedilol permeated at various time points is shown in Figure 3b. For both free drug and F18, the drug was not detectable within the first 6 h. At 20 and 24 h, carvedilol in F18 penetrated the skin to a slower degree than the free drug, consistent with its in vitro drug release profile (Figure 1c). At 24 h, F18 and the free drug showed 14.3 ± 6.71% (570 ± 268 ng) and 22.3 ± 9.11% (894 ± 365 ng) permeation, respectively (*p* < 0.05, *n* = 6~10). At 24 h, the skin was collected and a tape-stripping technique was performed to analyze the carvedilol in the stripped skin (epidermis and dermis) (Figure 3c). For the free drug, 24.2 ± 4.57% (969 ± 183 ng) was found retained in the stripped skin, while for F18, 20.0 ± 5.93% (795 ± 237 ng) was found in the stripped skin. Although the skin retention data showed a lower trend for F18, no statistical difference was detected between the two groups (*p* > 0.05).

### 3.4. Intracellular Uptake of Fluorescent Dye-Labeled F18 by JB6 P+ Cells

**Figure 4:**
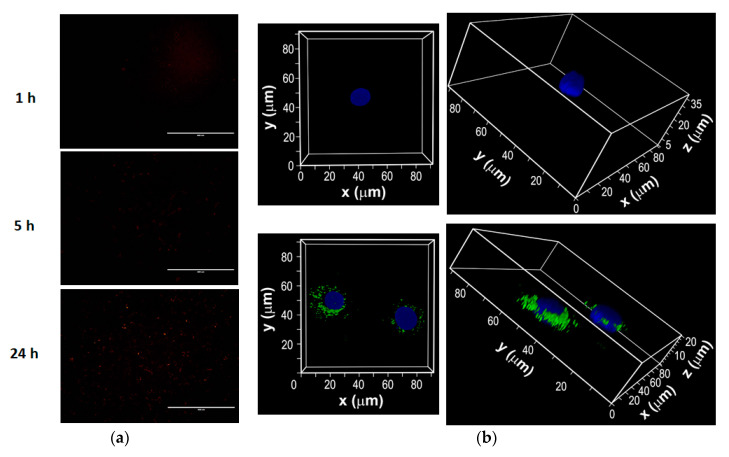
Intracellular uptake of DiI-labeled F18 by mouse epidermal JB6 P+ cells. (**a**) Epifluorescence images of cells incubated with DiI-labeled F18 for 1, 5, and 24 h. (**b**) Images were also taken with a laser scanning confocal microscope after the cells were incubated with F18 (labeled with DiI) for 3 h. Confocal Z-stacks of cells incubated with DiI-labeled F18 (green) for 3 h and Hoechst 33342 (blue). Upper panels: the control lacked drug treatment; lower panels: cells treated with F18. Magnifications are 63× by oil immersion. Overlay represents merged images from all channels.

In order to determine the effects of time on cellular uptake of F18, the formulation was prepared by incorporating the lipophilic fluorescent dye DiI. The JB6 P+ cells were incubated with labeled F18 for various time points. EVOS images showed that the internalization of the transfersome was time-dependent, as reflected by the greater intensity of red fluorescence after 24 h compared to earlier time points in Figure 4a. Since the EVOS image could not clearly show the intensity in time points earlier than 5 h, the experiments were repeated for the 3-h time point and images were taken by confocal microscopy, which confirmed the internalization of DiI labeled transfersome into cells (Figure 4b).

### 3.5. Cytotoxicity of F18 on JB6 P+ and HaCaT Cells

**Figure 5:**
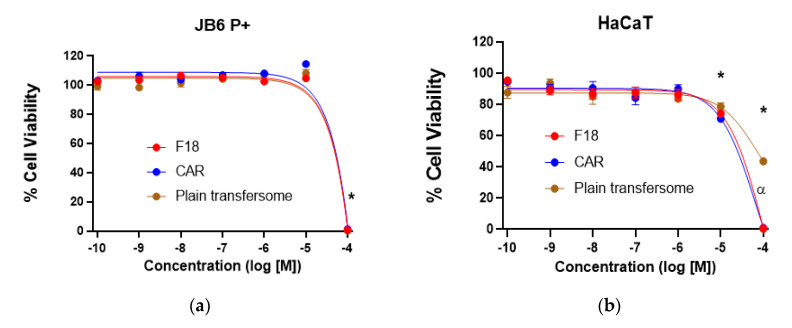

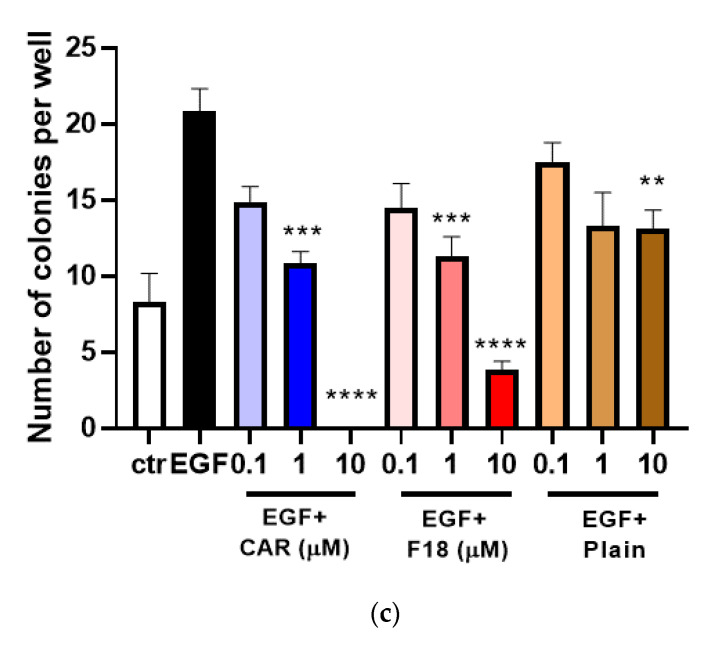
Effects of F18, free carvedilol, and the plain transfersome on cell viability of JB6 P+ and HaCaT cells and EGF-induced transformation of JB6 P+ cells. F18, free carvedilol, and the plain transfersome (with lipid concentrations equivalent to those in F18) were examined for their dose-dependent effects on the cytotoxicity (by SRB assay) on JB6 P+ (**a**) and HaCaT cells (**b**). Data were normalized to their respective controls. * *p* < 0.05 (2-factor ANOVA with Tukey Kramer post-hoc with an alpha set to 0.01). (**c**) Comparison of the effects of F18, free carvedilol, and the plain transfersome on the malignant transformation of JB6 P+ cells. The cancer preventive effects were examined using a soft agar assay. The data shown are the average numbers of colonies in each treatment group (*n* = 8). (** *p* < 0.01, *** *p* < 0.001, **** *p* < 0.0001).

Cell viability was determined for F18 in comparison with free carvedilol and the plain transfersome (at lipid concentrations equivalent to those in F18) after 72-h of incubation with JB6 P+ mouse epidermal cells and HaCaT human keratinocytes. In JB6 P+ cells, only the highest concentration (100 μM) was statistically different from all other concentrations tested. There was no biologically significant difference between the test agents at any given concentration (Figure 5a). In HaCaT cells, the highest concentrations (10 and 100 µM) were statistically different from all other concentrations tested; the plain transfersomes at 100 µM were significantly less toxic than F18 and free carvedilol (*p* < 0.01); all remaining data at any given concentration showed no biologically significant differences (Figure 5b). Although the SRB assay was conducted in vitro, these data indicate that F18 at concentrations higher than 10 µM may cause certain cytotoxicity. Thus, dose-dependent skin irritation and toxicity should be evaluated in human subjects in future studies.

### 3.6. Effects of F18 on EGF-Induced JB6 P+ Transformation

It has been demonstrated that carvedilol could inhibit the epidermal transformation induced by the tumor promoter EGF [3]. The EGF-induced colony formation assay on JB6 P+ cells was performed in soft agar using F18, free carvedilol, and the plain transfersome at equivalent lipid concentrations. As expected, cotreatment of EGF and free carvedilol of JB6 P+ cells inhibited colony formation induced by EGF in a dose-dependent manner (Figure 5c). Treatment of F18 showed a similar pattern of inhibition as free carvedilol: 1 and 10 μM F18 resulted in a significant reduction of colony numbers compared to the EGF only group. The colony numbers at the highest concentration of free carvedilol (10 μM) were zero, but treatment with F18 (10 μM) still resulted in some colonies, consistent with a slower drug release profile from F18. Treatment with plain transfersome showed a slightly reducing trend in colony numbers, and the highest dose (lipids concentration equivalent to 10 μM F18) was significantly different from other concentrations, indicating that the non-drug components of F18 partly contribute to the observed anti-transformation effects.

### 3.7. Drug Permeation of F18 in Full-Thickness 3D Human Reconstituted Skin Model

**Figure 6:**
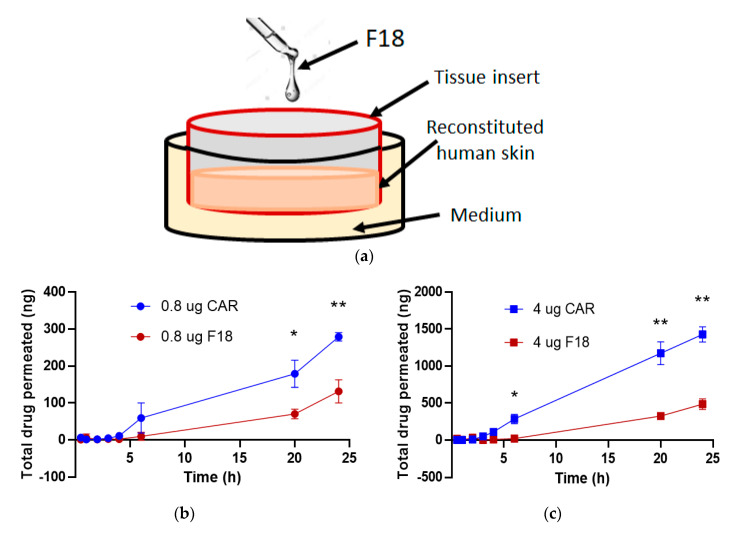
Carvedilol permeation in the reconstituted human 3D skin culture. (**a**) Schematic representation of Table 400. Treatment with F18 containing 0.8 μg (**b**), or 4 μg (**c**) carvedilol, or free drug of the same doses were applied to the surface of the skin culture and incubated for 24 h. Carvedilol levels within the medium under the membrane was measured as described in the Methods. Values are presented as the means ± SD of three wells (* *p* < 0.05, ** *p* < 0.01).

We applied free carvedilol in acetone or F18 to the SC surface of a reconstituted human full-thickness skin model (EpiDerm™ FT-400) (Figure 6a). This model contains both epidermal and dermal layers in 3D and has been reported to exhibit a similar response to UV as human skin [24]. The drug concentrations applied were 20 and 100 μM (equivalent to 0.8 and 4.0 μg of total drug amount), which showed protective effects on UV-induced damage of the same model in previous studies [5]. The reconstituted skin was cultured for 24 h after drug application, and at various time points, the levels of carvedilol in the medium under the membrane were measured. Carvedilol showed a time-dependent and concentration-dependent increase in skin permeation (Figure 6b,c). For free carvedilol, at 24 h, total carvedilol levels in the medium were 5.1-fold higher for a dose of 4.0 μg than 0.8 μg. F18 also showed similar dose-dependent permeation, and at 24 h, total carvedilol levels in the medium were 3.7-fold higher for a dose of 4.0 μg than 0.8 μg. For both doses, carvedilol levels in the medium were significantly higher for the free carvedilol than F18. At the 24-h time point, carvedilol levels in the medium when 0.8 μg F18 was applied were 47% of those treated with 0.8 μg free carvedilol (Figure 6b), while carvedilol levels in the medium when 4.0 μg F18 was applied were 34% of those treated with 4.0 μg free carvedilol (Figure 6c) (*p* < 0.05 for time points 20 and 24 h).

### 3.8. Effects of F18 on UV-Induced DNA Damage, Inflammation Markers and Apoptosis on Full-Thickness 3D Human Reconstituted Skin

**Figure 7:**
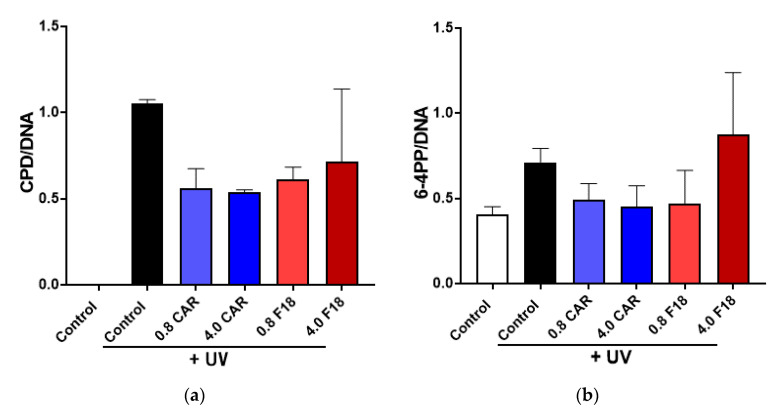
Effects of F18 on UV-induced CPD (**a**) and 6-4PP formation (**b**) in 3D human reconstituted skin. The skin was treated with UV radiation (300 mJ/cm^2^) with or without free carvedilol (CAR) or F18 in two doses for 24 h. Genomic DNA was isolated from the skin and analyzed with slot blot assays. Quantified and normalized CPD and 6-4PP values are expressed as mean + SE (*n* = 3).

Since drug permeation may result from damaged skin barrier, further studies are needed to determine acute and repeated dose toxicity of F18 and skin irritation on the same model. The 3D human reconstituted skin model, after being pretreated with drugs, was irradiated with 300 mJ/cm^2^ UV and cultured for an additional 24 h with carvedilol in acetone or F18 applied on the SC surface. UV radiation strongly induced the formation of DNA damage markers CPD and 6-4PP (Figure 7), which can be modestly attenuated by treatment with carvedilol (in acetone or F18) without statistical significance. Interestingly, 4.0 μg of F18 was less effective than the lower dose in both the CPD and 6-4PP assays. This result is consistent with a previous report where the effects of free carvedilol on DNA damage markers were modest in the in vitro assays [5].

**Figure 8:**
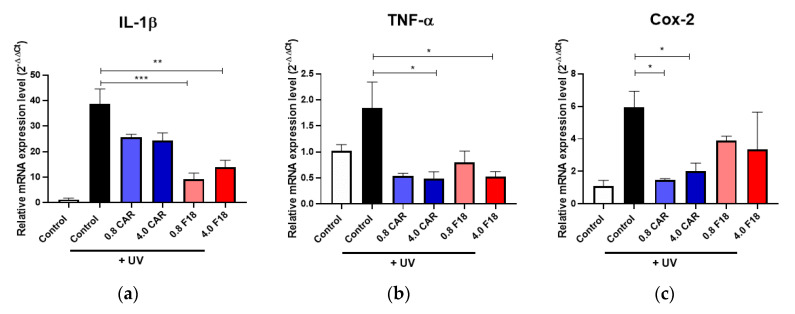
Effect of free drug or F18 on UV-mediated changes in the expression of proinflammatory genes IL-1β (**a**), TNF-α (**b**), and COX-2 (**c**) in 3D human reconstituted skin by qPCR. qPCR results from 3D culture treated by UV 300 mJ/cm^2^ and/or free carvedilol or F18. GAPDH was used as a normalization control for qPCR and the data are expressed as mean ± SE; *n* = 3. (***: *p* < 0.001; **: *p* < 0.01; *: *p* <0.05).

As expected, UV radiation also upregulated the expression of proinflammatory biomarkers including IL-1β, TNF-α, and COX-2, as detected by qRT-PCR (Figure 8). The qRT-PCR assay showed that F18 was able to attenuate these proinflammatory genes to a similar degree as carvedilol in acetone. Similar to the CPD/6-4PP assays, the inhibitory effects of F18 as well as the free drug were not dose dependent.

**Figure 9:**
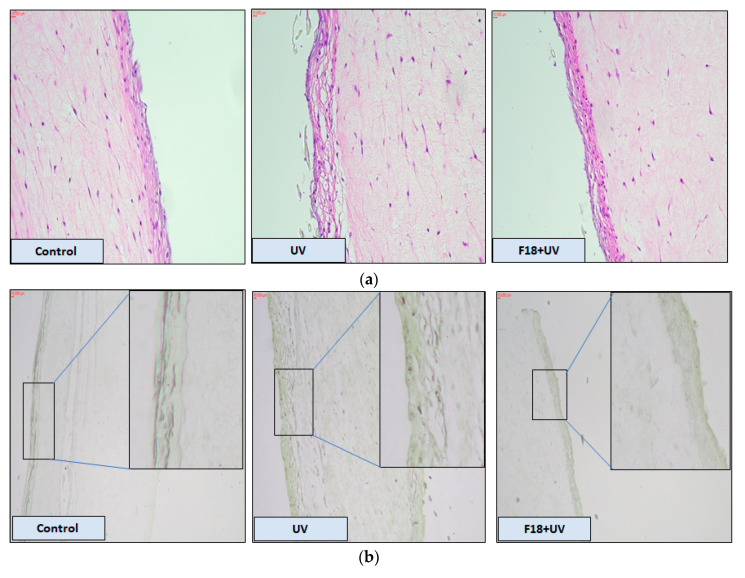
H&E staining and TUNEL assay in reconstituted human 3D skin model. (**a**) H&E staining showing morphological changes after the skin culture was treated with control, UV alone or F18 combined with UV. Magnification: 20×. (**b**) TUNEL assay demonstrated UV induced apoptosis can be attenuated by F18 treatment. Enlarged images emphasize apoptotic positive cells (magnification 10×). Scale bars, 10 μm.

The effect of F18 on 3D human reconstituted skin in terms of histological alteration caused by UV exposure is shown in Figure 9a. H&E staining showed that UV radiation led to considerable increase in epidermal thickening and damage compared to the control epidermis. Pre-treatment with F18 attenuated UV induced hyperplasia and skin damage. In Figure 9b, UV radiation increased the number of apoptotic cells (i.e., TUNEL positive cells) compared with the control without UV exposure. Following treatment with 4 µg F18, the number of TUNEL positive cells was reduced when compared with the UV only group.

## 4. Conclusions

Topical drug delivery offers many advantages for skin cancer chemoprevention, particularly for carvedilol, which is a highly potent β-blocker. Systemic absorption of carvedilol following oral administration may result in cardiovascular disturbance, which is undesirable as a cancer preventative agent. To repurpose carvedilol for skin cancer prevention, a novel topical formulation was developed in the present study for delivery of carvedilol directly to the skin with the goal of maintaining or enhancing efficacy, but mitigating systemic effects. All previous work related to carvedilol loaded topical delivery systems was to enhance systemic absorption for the treatment of hypertension [25,26,27]. This study, for the first time, demonstrated the feasibility of using the transfersomal delivery system for skin targeted delivery of carvedilol. Since this delivery system was designed for topical delivery, the ideal topical formulation should show a slower drug release for a sufficient time to avoid frequent application and to reduce systemic absorption. Carvedilol loaded transfersomes were prepared with various combinations of commonly used lipids and surfactants. Among the 30 carvedilol loaded transfersomes developed and characterized in terms of particle size, encapsulation, and zeta potential, one formulation, F18, was selected because it was the only one that consistently maintained small particle size with high stability. Franz diffusion cells and a porcine ear skin model proved that F18 was effective in the penetration and deposition of the drug in the skin, although at a slower rate compared to the free form of carvedilol dissolved in acetone. Studies on a 3D reconstituted human skin model confirmed that topical F18 produced photoprotective effects against UV-induced DNA damage, inflammation, and apoptosis. Therefore, a novel transfersomal delivery system for carvedilol was developed for the deposition of carvedilol into skin layers, which can achieve local pharmacological effects in the skin microenvironment while avoiding systemic side effects. Since the stability study was conducted at 4 °C, further investigation should evaluate the stability at room temperature. Furthermore, future studies will be directed to the development of sunscreen in the form of a gel or cream containing optimized carvedilol loaded transfersome, or by incorporating the transfersome into commercial sunscreen products. In vivo studies are necessary before we can suggest how carvedilol loaded transfersomes can be used to prevent skin cancer.

## Figures and Tables

**Table 1:** Physical characteristics of carvedilol-loaded transfersomes.

		Lipid	SA	Ratio	Size (nm)	PDI	Zeta Potential (mV)	EE (%)
Group 1	F1	DSPC	T-80	1:1:0.25	313.13 ± 3.26	0.28 ± 0.03	−5.54 ± 1.45	99.8
	F2	DSPC	T-80	1:2:0.25	341.65 ± 18.27	0.29 ± 0.02	−3.20 ± 0.19	99.4
	F3	DSPC	T-80	1:3:0.25	341.40 ± 19.77	0.23 ± 0.00	−2.35 ± 0.60	99.2
	F4	DSPC	T-80	1:4:0.25	619.14 ± 54.66	0.30 ± 0.02	−2.16 ± 0.19	99.6
	F5	DSPC	T-80	1:5:0.25	639.15 ± 73.71	0.26 ± 0.02	−1.89 ± 0.24	99.5
	F6	DSPC	T-80	1:1:0.5	370.11 ± 6.63	0.30 ± 0.01	−5.10 ± 0.56	100.8
	F7	DSPC	T-80	1:2:0.5	319.73 ± 10.37	0.28 ± 0.02	−2.06 ± 0.19	99.1
	F8	DSPC	T-80	1:3:0.5	246.26 ± 10.78	0.28 ± 0.01	−1.85 ± 0.06	98.5
	F9	DSPC	T-80	1:4:0.5	364.77 ± 18.35	0.29 ± 0.01	−1.86 ± 0.39	101.1
	F10	DSPC	T-80	1:5:0.5	365.96 ± 16.11	0.25 ± 0.01	−1.61 ± 0.30	100.7
Group 2	F11	SPC	T-80	1:1:0.25	286.92 ± 1.52	0.29 ± 0.01	11.60 ± 0.93	99.6
	F12	SPC	T-80	1:2:0.25	234.56 ± 3.37	0.27 ± 0.00	10.58 ± 1.42	97.2
	F13	SPC	T-80	1:3:0.25	213.46 ± 1.46	0.28 ± 0.01	12.53 ± 1.15	91.6
	F14	SPC	T-80	1:4:0.25	238.50 ± 3.14	0.28 ± 0.01	13.3 ± 0.96	100.3
	F15	SPC	T-80	1:5:0.25	240.41 ± 3.63	0.27 ± 0.01	13.07 ± 0.53	91.9
	F16	SPC	T-80	1:1:0.5	243.73 ± 37.47	0.30 ± 0.05	8.74 ± 0.24	99.5
	F17	SPC	T-80	1:2:0.5	204.55 ± 41.88	0.24 ± 0.03	15.13 ± 1.06	98.9
	F18	SPC	T-80	1:3:0.5	162.92 ± 9.57	0.26 ± 0.01	17.13 ± 0.29	99.8
Group 3	F19	HEPC	T-80	1:1:0.25	301.47 ± 7.33	0.25 ± 0.03	−3.54 ± 0.88	98.8
	F20	HEPC	T-80	1:2:0.25	380.62 ± 32.01	0.25 ± 0.03	−1.01 ± 0.30	98.8
	F21	HEPC	T-80	1:3:0.25	214.38 ± 5.31	0.26 ± 0.01	−2.85 ± 1.11	98.8
	F22	HEPC	T-80	1:1:0.5	477.29 ± 57.66	0.32 ± 0.02	−1.81 ± 0.28	98.1
	F23	HEPC	T-80	1:2:0.5	272.68 ± 6.52	0.32 ± 0.01	−1.11 ± 0.40	97.7
	F24	HEPC	T-80	1:3:0.5	381.12 ± 70.36	0.28 ± 0.02	−1.20 ± 0.34	99.7
Group 4	F25	SPC	SC	1:1:0.25	303.63 ± 2.90	0.27 ± 0.01	8.14 ± 0.81	97.6
	F26	SPC	SC	1:2:0.25	224.96 ± 2.62	0.26 ± 0.01	12.13 ± 0.24	98.8
	F27	SPC	SC	1:3:0.25	213.14 ± 1.49	0.26 ± 0.01	12.33 ± 0.47	94.1
	F28	SPC	SC	1:1:0.5	206.41 ± 5.51	0.30 ± 0.01	9.62 ± 0.19	96.9
	F29	SPC	SC	1:2:0.5	217.44 ± 5.13	0.27 ± 0.01	12.53 ± 0.44	98.2
	F30	SPC	SC	1:3:0.5	189.12 ± 2.23	0.27 ± 0.01	12.43 ± 0.28	91.9

Ratio = carvedilol:lipids:surfactant. SA: surfactant. PDI: polydispersity index. T-80: Tween 80. SC: sodium cholate, EE: encapsulation efficiency. Volume of formulation: 10 mL. Final drug concentration: 0.1 mg/mL.

**Table 2:** Physical characteristics of the carvedilol-loaded transfersomes.

	Lipid	SA	Ratio	Size (nm)	PDI	Zeta Potential (mV)	EE%
F8	DSPC	T-80	1:3:0.5	549.97 ± 25.11	0.31 ± 0.01	0.9 ± 0.1	53.9
F18	SPC	T-80	1:3:0.5	197.11 ± 4.68	0.3 ± 0.01	15.7 ± 0.7	69.7
F21	HEPC	T-80	1:3:0.25	2164.57 ± 162.45	0.34 ± 0.02	−0.5 ± 0.8	93.1
F30	SPC	SC	1:3:0.5	217.69 ± 3.8	0.3 ± 0.01	21 ± 0.9	46.1

Ratio = carvedilol:lipids:surfactant. SA: surfactant. PDI: polydispersity index. T-80: Tween 80. SC: sodium cholate, EE: encapsulation efficiency. Volume of formulation: 10 mL. Final drug concentration: 0.5 mg/mL.
